# Embolization in Patient with Hypovolemic Shock after Transobturator Sling Procedure

**DOI:** 10.1055/s-0040-1718435

**Published:** 2020-11-30

**Authors:** Germano José Ferraz de Arruda, Miguel Bonfitto, Jerônimo Ferraz de Arruda Neto, Luis Cesar Fava Spessoto, José Germano Ferraz de Arruda, Fernando Nestor Fácio

**Affiliations:** 1 Faculdade de Medicina de São José do Rio Preto, São José do Rio Preto, SP, Brazil; 2 Hospital São Domingos, Catanduva, SP, Brazil

**Keywords:** hemorrhagic shock, embolization, postoperative complications, suburethral sling, urinary incontinence, choque hemorrágico, embolização, complicações pós-operatórias, sling, incontinência urinária

## Abstract

The placement of a suburethral sling is standard treatment for stress urinary incontinence. The transobturator technique (TOT) emerged as an alternative to minimize the risks of the blind insertion of needles, leading to a lower rate of perforation complications compared with the retropubic approach. We present a case of injury to a branch of the left obturator artery following the placement of a urethral sling using TOT, followed by intense bleeding and hemodynamic instability, which was treated with embolization.

## Resumo

Sling de uretra média é o tratamento padrão para a incontinência urinária de esforço. A abordagem transobturatória (TOT) surgiu como alternativa para minimizar os riscos da inserção às cegas das agulhas com taxa de complicações perfurativas menores quando comparadas à abordagem retropúbica. Apresentamos um caso de lesão em ramo da artéria obturatória esquerda após sling TOT que evoluiu com sangramento intenso e instabilidade hemodinâmica, sendo tratado com embolização.

## Introduction

Urinary incontinence is defined as the involuntary loss of urine that may or may not be associated with stress and, according to the International Continence Society, stress urinary incontinence is that caused by coughing, sneezing, laughing, jumping or exercise. 1 The placement of a mid-urethral sling is the standard treatment option for this condition, and the guidelines of the American Urological Association state that this procedure is the most widely studied, with follow-up for up to 15 years. 2 Sling placement is used due to the ease of implantation, fast recovery, low morbidity rate and a middle- and long-term cure rate comparable to that reported for well-established surgical treatments, such as the Burch colposuspension and pubovaginal sling procedures. 3

Despite its efficacy and safety, such procedures can be associated with complications. Hemorrhage with blood loss > 200 mL or postoperative hematoma occur in ∼ 2% of patients and can be controlled with compression or rest alone. 4 Injury to larger vessels are rare, occurring in 0.07% of cases. However, when there is the suspicion of a vessel injury, especially with hemodynamic instability, primary surgical repair is required. 5 To reduce complications stemming from sling placements, Delorme 6 presented the transobturator technique (TOT) as a surgical alternative, in which the needle is passed through the obturator foramen without reaching the retropubic space, thereby minimizing the risk of complications, such as bleeding, which is rarely described in TOT. 6 7

We present the case of a patient submitted to sling placement with TOT that evolved with hypovolemic shock in the postoperative period, which was treated with embolization.

## Case Description

The present report involves a 54-year-old woman with a history of arterial hypertension, the use of a β-blocker, dyslipidemia and fibromyalgia, no history of surgeries and a complaint of stress urinary incontinence for ∼ 5 years. The physical examination revealed no prolapse of the pelvic organs. The urodynamic test showed urinary incontinence upon effort with low pressure and no evidence of involuntary detrusor contractions.

**Fig. 1:**
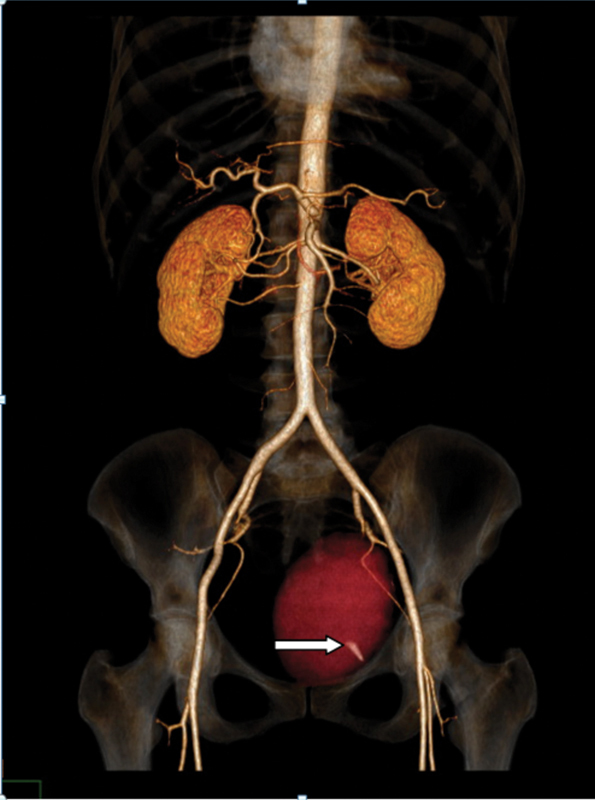
Tomogram showing pelvic hematoma on left (in red). White arrow indicates active bleeding posterior to the obturator foramen.

**Fig. 2:**
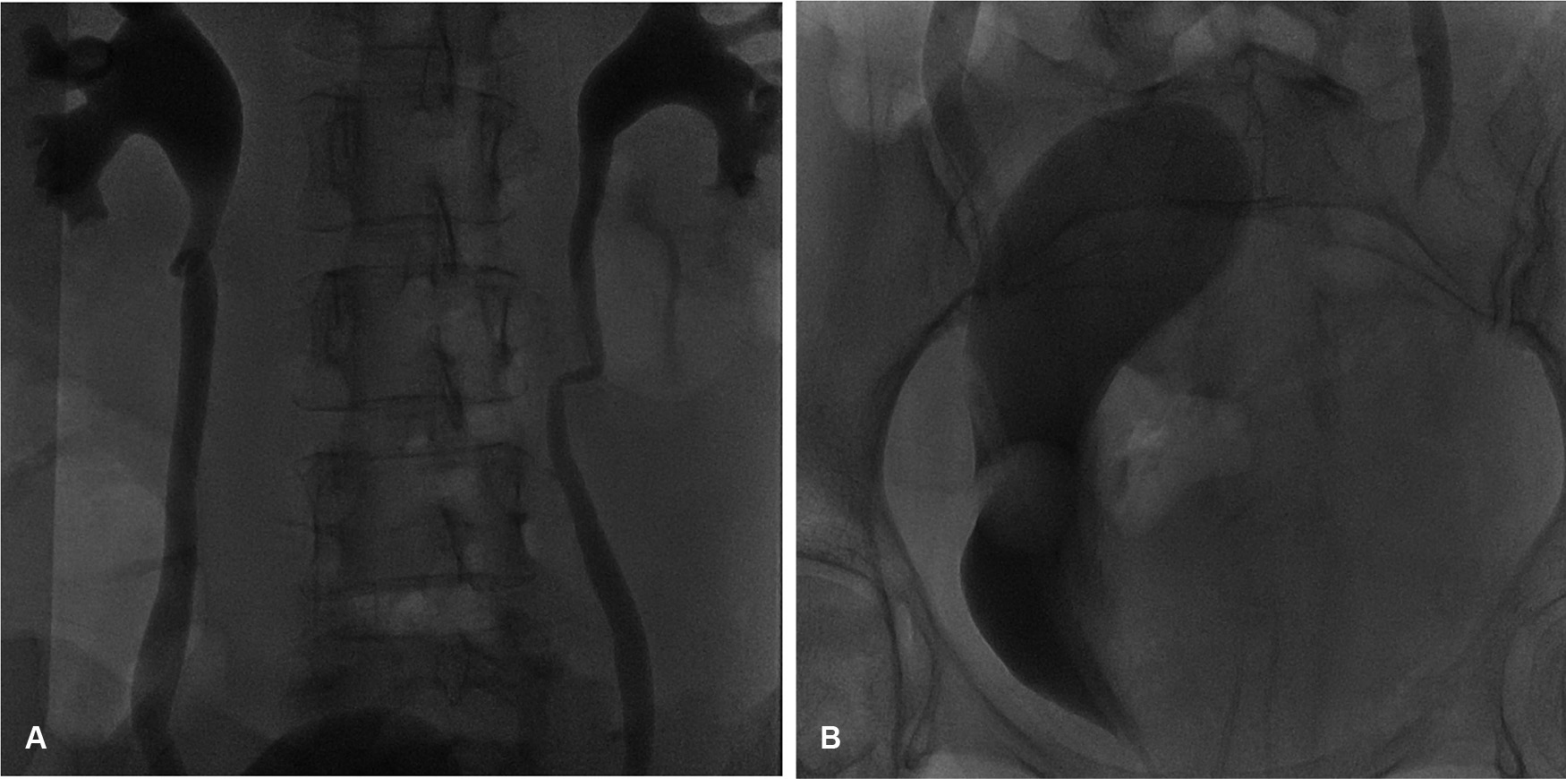
X-ray angiography showing (A) bilateral dilation of the pyelocaliceal system and (B) bladder displaced to the right.

**Fig. 3:**
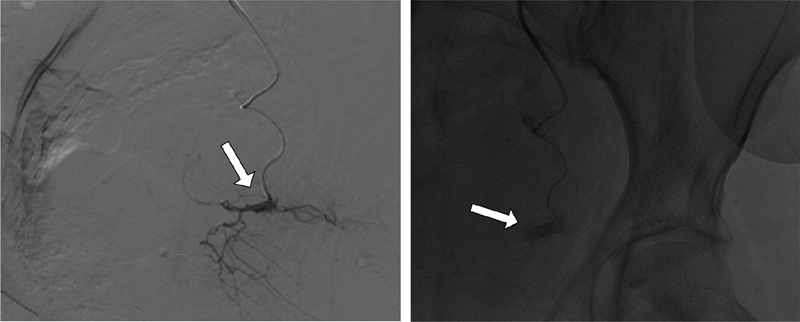
Arteriogram with leakage of contrast (white arrow) in a branch of left obturator artery, revealing active bleeding.

The patient was submitted to the placement of a urethral sling using TOT, with no complications during the procedure, and remained asymptomatic during anesthetic recovery. Four hours after surgery, the patient complained of pain in the lower region of the abdomen and abdominal distention. She presented hypovolemic shock (blood pressure: 70 × 40 mm Hg) and tachycardia (heart rate: 96 bpm), with no vaginal bleeding or evident abdominal or perineal hematoma. After fluid resuscitation with crystalloids, the patient was submitted to a computed tomography (CT), which revealed a left peribladder hematoma (12.4 × 7.9 × 8.9 cm) and active bleeding in a branch of the left obturator artery ( Fig. 1 ). X-ray angiography revealed bilateral dilation of the pyelocaliceal system and bladder displaced to the right ( Figs. 2A and 2B ). The exam confirmed the tomographic finding with regards to active bleeding ( Fig. 3 ), which ceased after embolization. The patient remained in intensive care for 2 days and received a red blood cell transfusion, after which the red series remained stable. She was discharged on the 4 ^th^ day after the surgical procedure. The follow-up CT in 6 months revealed that the hematoma was in regression. The patient reported being satisfied with the results of the procedure, despite the complication.

## Discussion

When conservative treatment for urinary incontinence fails, midurethral sling is a therapeutic option. The TOT emerged as an alternative with a lower morbidity rate compared with the retropubic technique. However, post-TOT complications can occur in rare cases, such as bladder perforation, urinary retention, pelvic hematoma, infection, erosion/extrusion and pain. 8

Large series and meta-analyses have demonstrated a lower risk of hemorrhagic complications with TOT. Bleeding rates range from 0.7 to 8% with the retropubic technique and from 0 to 2% with TOT. 9 10 Reviewing patients between 2001 and 2005, Deng et al 11 found that only 0.1% required blood transfusions. Normally, such complications do not evolve to hypovolemic shock and are treated conservatively. 6

In the present report, we describe a rare case of a patient submitted to TOT that evolved in the postoperative period to hypovolemic shock and was treated with embolization. After stabilization, the patient was submitted to arteriography, which revealed bleeding from a branch of the left obturator artery. The present study is important due to the fact that the patient did not exhibit perineal ecchymosis or vaginal bleeding; the suspicion of the complication was based on the rapid evolution to hemodynamic instability. Moreover, the fact that we are a tertiary hospital with available CT and an angiography service with embolization contributed to the nonsurgical treatment of this serious complication and a satisfactory outcome.

## Conclusion

In the present report, the outcome of endovascular treatment was satisfactory, leading to hemodynamic stability and the non-need for surgical intervention. As a rare occurrence, there are few reports of the use of embolization for the treatment of post-TOT bleeding. It is important for surgeons to be aware of this possibility and the available therapeutic arsenal to avoid surgical reintervention.

## References

[1] Standardisation Sub-Committee of the International Continence Society Abrams P Cardozo L Fall M Griffiths D Rosier P Ulmsten U The standardisation of terminology in lower urinary tract function: report from the standardisation sub-committee of the International Continence Society Urology 2003 61 01 37 49 10.1016/s0090-4295(02)02243-4 12559262

[2] Kobashi K C Albo M E Dmochowski R R Ginsberg D A Goldman H B Gomelsky A Surgical treatment of female stress urinary incontinence: AUA/SUFU guideline J Urol 2017 198 04 875 883 10.1016/j.juro.2017.06.061 28625508

[3] Lee D Bacsu C Dillon B Zimmern P E Complications following the insertion of two synthetic mid-urethral slings and subsequent removal Low Urin Tract Symptoms 2018 10 03 259 265 10.1111/luts.12175 28657139

[4] Cetinel B Tarcan T Management of complications after tension-free midurethral slings Korean J Urol 2013 54 10 651 659 10.4111/kju.2013.54.10.651 24175037 PMC3806987

[5] Sivanesan K Abdel-Fattah M Ghani R External iliac artery injury during insertion of tension-free vaginal tape: a case report and literature review Int Urogynecol J Pelvic Floor Dysfunct 2007 18 09 1105 1108 10.1007/s00192-006-0283-7 17221147

[6] Delorme E [Transobturator urethral suspension: mini-invasive procedure in the treatment of stress urinary incontinence in women] Prog Urol 2001 11 06 1306 1313 11859672

[7] Cho E J Kim J B Park S Y Kim S H Kim C H Kang B M Chae H D Pelvic artery embolization in the management of pelvic arterial bleeding following midurethral sling surgery for stress urinary incontinence Obstet Gynecol Sci 2016 59 02 163 167 10.5468/ogs.2016.59.2.163 27004210 PMC4796089

[8] Zhang P Fan B Zhang P Han H Xu Y Wang B Zhang X Meta-analysis of female stress urinary incontinence treatments with adjustable single-incision mini-slings and transobturator tension-free vaginal tape surgeries BMC Urol 2015 15 64 10.1186/s12894-015-0060-3 26148987 PMC4492097

[9] Gomes C M Carvalho F L Bellucci C HS Hemerly T S Baracat F de Bessa J Jr Update on complications of synthetic suburethral slings Int Braz J Urol 2017 43 05 822 834 10.1590/S1677-5538.IBJU.2016.0250 28266818 PMC5678512

[10] Porena M Costantini E Frea B Giannantoni A Ranzoni S Mearini L Tension-free vaginal tape versus transobturator tape as surgery for stress urinary incontinence: results of a multicentre randomised trial Eur Urol 2007 52 05 1481 1490 10.1016/j.eururo.2007.04.059 17482343

[11] Deng D Y Rutman M Raz S Rodriguez L V Presentation and management of major complications of midurethral slings: Are complications under-reported? Neurourol Urodyn 2007 26 01 46 52 10.1002/nau.20357 17149713

